# Dissociating Cortical Activity during Processing of Native and Non-Native Audiovisual Speech from Early to Late Infancy

**DOI:** 10.3390/brainsci4030471

**Published:** 2014-08-11

**Authors:** Eswen Fava, Rachel Hull, Heather Bortfeld

**Affiliations:** 1 Department of Psychology, University of Massachusetts Amherst, 135 Hicks Way, Amherst, MA 01003, USA; 2 Department of Psychology, Texas A&M University, 4235 TAMU, College Station, TX 77845, USA; E-Mail: rhull@tamu.edu; 3 Department of Psychology, University of Connecticut, 406 Babbidge Road, Unit 1020, Storrs, CT 06269-1020, USA; E-Mail: heather.bortfeld@uconn.edu; 4 Haskins Laboratories, 300 George St #900, New Haven, CT 06511, USA

**Keywords:** near-infrared spectroscopy (NIRS), perceptual narrowing, infancy, audiovisual speech perception, language, language development, speech perception

## Abstract

Initially, infants are capable of discriminating phonetic contrasts across the world’s languages. Starting between seven and ten months of age, they gradually lose this ability through a process of perceptual narrowing. Although traditionally investigated with isolated speech sounds, such narrowing occurs in a variety of perceptual domains (e.g., faces, visual speech). Thus far, tracking the developmental trajectory of this tuning process has been focused primarily on auditory speech alone, and generally using isolated sounds. But infants learn from speech produced by people talking to them, meaning they learn from a complex audiovisual signal. Here, we use near-infrared spectroscopy to measure blood concentration changes in the bilateral temporal cortices of infants in three different age groups: 3-to-6 months, 7-to-10 months, and 11-to-14-months. Critically, all three groups of infants were tested with continuous audiovisual speech in both their native and another, unfamiliar language. We found that at each age range, infants showed different patterns of cortical activity in response to the native and non-native stimuli. Infants in the youngest group showed bilateral cortical activity that was greater overall in response to non-native relative to native speech; the oldest group showed left lateralized activity in response to native relative to non-native speech. These results highlight perceptual tuning as a dynamic process that happens across modalities and at different levels of stimulus complexity.

## 1. Introduction

It is generally accepted that infants learn language with relative ease. They are capable of seemingly extraordinary feats, such as extracting “words” from a continuous stream of speech based on statistical structure alone [1], or differentiating between two languages given only visual access to the person speaking to them [2]. In the daily life of a typically developing infant, however, information about language is available in the form of richly varied audiovisual speech. From this input, they perform another amazing feat: by the end of the first year of life, they perceptually tune to the sounds of their native language (or languages) and consequently lose sensitivity to other languages’ sounds [3,4]. This phenomenon has served as the foundation for the theoretical framework known as perceptual narrowing, or tuning. In recent years, many forms of such tuning have been documented (for a review, see [5]), among them perceptual tuning to audiovisual speech [6].

Perceptual tuning—and early language development more generally—takes place in the context of both speech input and brain maturation. There is substantial debate about the relative contribution of each to the learning process. Much of the data concerning changes in infants’ sensitivity to speech are behavioral in nature, and are therefore limited in what they can tell us about the specific neural processes underlying those changes. In recent years, the application of neurophysiological techniques has advanced our understanding of the relationship between infants’ behavioral responses to speech and the neural mechanisms that support its processing [7]. As such, neurophysiological measures should be ideal for studying the interface between early brain development and environmental experience, and therefore the process of perceptual tuning.

The original findings on phonetic sensitivity showed that infants begin life capable of discriminating between all phonetic contrasts [8,9]. Subsequently, it was revealed that this discrimination profile narrows before the end of the first year to exclude non-native language contrasts [10]. In order to more specifically examine the progression of native language tuning, behavioral researchers have examined several types of phonetic contrasts and, while finding exceptions in the overall timeline (e.g., [11,12]) likely due to the relative difficulty or “acoustic salience” of particular phonemes [13], there is a general consensus that infants become “tuned” to their native phonemic inventory by the beginning of their second year [14,15,16,17]. In short, behavioral evidence converges on the second half of the first year of life (in particular between 7 and 10 months) as the time when this tuning occurs, at least in monolingually-exposed infants. Although sensitivity to other aspects of speech likewise tunes, this happens at different points in early development depending on the specific characteristics of the speech cue in question. Indeed, preference for native speech relative to speech from languages with dissimilar rhythmic structures has been observed immediately after birth [18,19,20]. This initial preference likely stems from substantial prenatal experience, given the low-pass filtering effect the prenatal environment has on speech [20].

Tuning continues postnatally. In their first six months of life, infants shift from a predominantly suprasegemental representation of speech [21] to a more refined representation based on additional features of the signal [22,23]. For example, by five months, infants can discriminate among languages from the same stress class [24], presumably positioning them for the phoneme-specific sensitivity that emerges in the second half of the first year. At the same time, sensitivity to other perceptual forms emerges. Among other things, infants become increasingly sensitive to faces from their own *versus* other species, from their own *versus* other ethnic groups [25,26], to familiar *versus* unfamiliar forms of visual speech [2], and to audiovisual speech itself [6]. However, despite substantial behavioral evidence, clear neural markers of these transitions remain elusive.

Potential candidates do exist. For example, electrophysiological evidence of prosody-specific processing in four-month-olds has localized cortical activity to right temporal regions [27]. This was observed while infants listened to single words with contrasting (native *versus* non-native) stress patterns (e.g., /papa/ and /papá/). Importantly, such data have been obtained more broadly using near-infrared spectroscopy (NIRS) [28,29] and the lasting nature of this processing bias has been confirmed using functional magnetic resonance imaging (fMRI) in both children [30,31] and adults [32,33,34], highlighting one domain of perceptual tuning for which behavioral and neurophysiological measures cohere. Unfortunately, determining whether the right hemisphere localization of prosody-evoked cortical activity is present from birth or is a shift that occurs in the first months of life has been harder to determine. This is the case for other aspects of speech as well. Despite a variety of behavioral and neurophysiological studies (for reviews, see [5,35]), the nature of the specific questions and constraints inherent in testing any particular infant age group have prevented access to easily comparable findings, even within a single methodological approach.

If we focus just on results obtained using NIRS with infants, we see that investigators have utilized several different types of stimuli representative of the different components of the speech signal (e.g., single phonemes, CV syllables, words, and sentences) whose influence is of interest. Briefly, sentence-level speech stimuli are used when investigators are interested in infants’ emerging sensitivity to suprasegmental (e.g., prosodic) cues. One example of this is the examination of how neonates and older infants process their native language when it is delivered in infant-directed speech (IDS) or adult-directed speech (ADS). Typically, IDS consists of more variable pitch, a higher overall fundamental frequency, more repetition and simpler sentence structure when compared with ADS, features that make it more engaging and thus facilitative of early learning [36,37,38,39]. The stimuli used in neurophysiological studies tend to be auditory-only, continuous speech in one of the two forms (IDS *versus* ADS). The cortical sites that differentially process auditory-only IDS and ADS shift across the course of the first year, with neonates showing differential cortical activity in response to these two types of stimuli in the frontal regions (located under 10-20 sites Fp_1_, Fp_2_) [40] and older infants showing it in bilateral temporal areas [41].

Another approach is to compare infants’ processing of different aspects of familiar (e.g., native) *versus* unfamiliar (e.g., non-native) speech. Not surprisingly, these also reveal substantial differences in cortical responses as a function of experience. For example, in our own work using continuous speech, we found that 6-to-9-month-old infants showed a left lateralized response to continuous native speech [42]. A left lateralized pattern was also observed in neonates in response to continuous native speech presented running forward relative to backwards [43,44]. Japanese-exposed neonates likewise showed a left lateralized temporoparietal pattern of activation in response to continuous forward compared to backward Japanese, while no differential processing was observed for the same contrast in another language [44]. Taken together, these findings support the widely accepted view that early processing of continuous native speech is left lateralized, both in neonates and in older infants. However, any further regional specificity of these claims is limited by the different stimuli and age groups tested.

Cortical processing patterns emerge at different ages in response to isolated speech sounds (e.g., CV syllables, words) as well. For example, Minagawa-Kawaii and colleagues [45] measured cortical activity in bilateral temporal regions while exposing several age groups of infants (3-to-4 months, 6-to-7 months, 10-to-11 months, 13-to-14 months, 25-to-28 months) to pseudowords whose final vowel duration varied along a continuum (e.g., /mama/ and /mama:/). A within-category contrast represented a non-native language phonemic boundary, and an across-category contrast represented a native language phonemic boundary. The researchers found that the 3-to-4- and 10-to-11-month-olds showed no differences in cortical activation in response to the two stimulus types. In contrast, the 6-to-7, 13-to-14, and 25-to-28-month-olds showed greater overall activity in response to the across-category contrast, and only the latter two age groups showed specifically left lateralized activation in response to it (but for contrasting results with low-pass filtered backward and forward native and non-native sentence-level speech, see [46]). In addition, Petitto and colleagues [47] exposed monolingual infants (4 and 12 months of age) to consonant-vowel syllables with native and non-native phonetic units. They found that 12-month-old and not 4-month-old infants showed robust activation in the left inferior frontal cortex (IFC) to only native (not non-native) stimuli. Thus, in contrast to the age-wide differences observed in response to continuous speech [42,43,44], more isolated segments of speech elicited differential responses only in older infants [45,47].

In another study, researchers compared cortical responses within an age group (*i.e.*, newborns) to different linguistic features. Specifically, when responses to a specific prosodic contrast (e.g., the prosodically distinct word pairs /itta/ *versus* /itta?/ in Japanese) were compared to a specific phonological contrast (/itta/ *versus* /itte/), neonates showed greater activity in the right temporal cortex relative to the left in response to the former and in bilateral temporal regions in response to the latter [48]. Thus, different classes of speech cues (*i.e.*, prosodic, phonological) engage the infant brain differently. Moreover, whether or not auditory stimuli are presented along with visual stimuli seems to influence processing. In a study in which Minagawa-Kawai and colleagues [49] exposed 4-month-olds to continuous native and non-native speech while they were also engaged with toys, the 4-month-olds showed a left lateralized response to native compared with non-native speech in the left temporal area. These data highlight the fact that focal regions of cortical activity in response to any particular stimulus shift substantially across the first year of life.

Overall, these results underscore the importance of considering the nature of the stimuli themselves in the interpretation of results. Given the range of possible stimulus manipulations (e.g., native *versus* non-native speech; smaller *versus* larger components of speech; auditory speech with or without visual stimuli; visual stimuli that are or are not explicitly related to the auditory stimuli), it is clear that the source of variability in cortical processing patterns is as likely to be the structure of the stimuli as it is to be the age of the infant.

What emerges from the research reviewed thus far is that the richer a stimulus contrast is, the younger the age at which processing differences can be observed in response to it [5]. Moreover, visual stimuli engage infants’ attention in a way that auditory-only speech does not. Because infants can discriminate between native and non-native visual speech (that is, without the accompanying audio) [2], adding the visual component to auditory speech may further heighten infants’ engagement with the stimuli [50].

The goal of the current study was to identify changes in patterns of neural activity while infants of different ages were exposed to ecologically coherent (e.g., audiovisual) familiar (native) and unfamiliar (non-native) continuous speech. Because we used continuous speech with accompanying visual speech, we predicted that even very young infants would show evidence of differential cortical activation in response to native and non-native speech. In addition, the richness of the stimuli should allow maximal dissociation of processing regions in infants as they get older and continue to gain experience with their native (in this case, English) language.

## 2. Experimental Section

### 2.1. Participants

Participants were 35 infants (17 females; between the ages of 3 and 14 months). Fourteen 3-to-6-month-olds were tested (mean age 167 days, 7 females); thirteen 7-to-10-month-olds were tested (mean age of 252 days, 6 females), and eight 11-to-14-month-olds were tested (mean age of 352 days, 4 females). Infants’ names were obtained from birth announcements in the local newspaper and commercially produced lists, and infants and parents were offered a new toy as compensation for their participation. Infants were all from monolingual English speaking households and caretakers verified that they were not exposed to Spanish in their day-to-day lives.

Informed consent was obtained from parents before testing began. An additional ten infants were tested, but their data were not included in the final analyses for the following reasons: five did not contribute enough data to be included in our sample, either due to excessive crying or to removing the NIRS probe during experimentation; data from four were lost due to machine malfunction during the testing; and one infant moved excessively during testing, rendering the data unanalyzable.

### 2.2. Stimuli and Design

The stimuli consisted of ten audiovisual 20-second-long, child-friendly stories, delivered in infant directed style speech by a bilingual female speaker in English (native condition) or Spanish (non-native condition). Five trials were delivered in English and five trials were delivered in Spanish (the order was counterbalanced across subjects). The same speaker was used for both Spanish and English to control for influences of speaker identity, which has been shown to play a role in early speech processing [51]. Spanish and English were used since they are not maximally contrastive in prosodic form, but are from different stress families [52].

**Figure 1:**
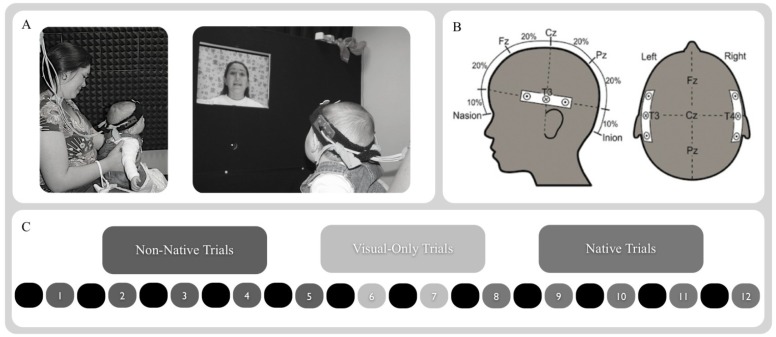
Methods. (**A**) Infants were exposed to speech stimuli while seated on a caregiver’s lap. (**B**) Infants wearing the NIRS headband, localized using the 10-20 coordinates T3 and T4. (**C**) Infants were tested using a block-design that consisted of 20 s long stretches of non-native and native infant-directed audiovisual speech. Each trial was preceded by a 10 s silent baseline. The two types of audiovisual speech block were further separated by two 20 s long trials of animated shapes with no accompanying audio.

Briefly, infants observed five trials of native or non-native speech, then two visual-only trials were played (animated shapes), then five trials of the alternate speech type were played. The stimuli were presented such that each trial was preceded by a 10 s baseline period, during which the screen was black and no sound was played. Each trial lasted for 20 s. We used a blocked design to maximize the changes in hemodynamic activity across the alternating perceptual events. The two 20 s visual-only trials were included between test blocks to further demarcate infants’ neural processing of native and non-native speech stimuli. These consisted of three-dimensional animated objects (e.g., spirals, circles, and rectangles) that were presented against a high-contrast, colored background with no accompanying sound (see Figure 1C). The animations were designed to be similar in color contrast and motion parameters using 3-D Studio Max™ computer graphics software.

### 2.3. Procedure

Infants were positioned on their caretaker’s lap facing a 53-cm flat panel computer monitor (Macintosh G4) 76 cm away (approximately 28.1° visual angle at infants’ viewing distance based on a 36-cm-wide screen). After the caretaker and infant were seated, a head circumference measurement was taken from the infant using a standard cloth tape measure and the 10-20 sites T_3_ position (on the left) and T_4_ position (on the right) were identified and marked on the child’s head with an erasable pen. The experimenter then placed the probe on the infant’s head, positioning it so that the two sets of channels were located over the left and right temporal areas, centered over T_3_ (on the left) and T_4_ (on the right) as established based on the head circumference measure. Caretakers were instructed to refrain from talking or interacting with infants during the course of the experiment, and to hold infants up so that they were able to comfortably view the screen. They were also asked to guide infants’ hands down and away from the headband if they began to reach up during the experiment. See Figure 1A for examples of infants in the testing booth during testing with the NIRS probe in place.

The experimenter then moved to the control area, lights in both the experimental and control areas were turned off, leaving only a low intensity light to illuminate the experimental area and light from the computer monitor to light the control area, lasers on the imaging device were turned on, and stimulus presentation and optical recordings began. Infants were video recorded for the duration of the session for later coding of looking behavior.

### 2.4. NIRS Probe and Apparatus

The NIRS instrument was developed by TechEn (Cambridge, MA, USA) and consisted of three major components: (1) two fiber optic cables that delivered near-infrared light to the scalp of the participant (*i.e.*, emitter fibers); (2) four fiber optic cables that detected the diffusely reflected light at the scalp and transmitted it to the receiver (*i.e.*, detector fibers); and (3) an electronic control box that served both as the source of the near-infrared light and the receiver of the refracted light. The signals received by the electronic control box were processed and relayed to a DELL Inspiron 7000™ laptop computer. A custom computer program recorded and analyzed the signal.

The imaging device used in these studies produced light at 680 and 830 nm wavelengths with two laser-emitting diodes [53]. The laser power emitted from the end of the fiber was 4 mW, and light was square wave modulated at audio frequencies of approximately 4 to 12 kHz. Each laser had a unique frequency so that synchronous detection could uniquely identify each laser source from the photodetector signal. Any ambient illumination that occurred during the experiment (e.g., from the visual stimuli) did not interfere with the laser signals because environmental light sources modulate at a significantly different frequency. No detector saturation occurred during the experiment. The light was delivered via fiber optic cables (*i.e.*, fibers), each 1 mm in diameter and 15 m in length. These originated at the imaging device and terminated in the headband that was placed on the infant’s head.

The headband was made of elastic terry-cloth and was fitted with the two light-emitting and four light-detecting fibers. These were grouped into two emitter/detector fiber sets (*i.e.*, optical probes), each containing two detector fibers placed at 2 cm distance on either side from the central emitter fiber. One optical probe was used to deliver near-infrared light to the left temporal region at approximately position T_3_ according to the International 10-20 system, and the other delivered light to the right temporal region at approximately position T_4_ according to the International 10-20 system.

### 2.5. Filtering and Motion Artifact Detection and Correction

The NIRS data were processed and analyzed for each neural area separately, using a procedure similar to that of Wilcox *et al.* [54]. Briefly, the raw signals were acquired at the rate of 200 samples per second, digitally low-pass- filtered at 10.0 Hz, a principal components analysis was used to design a filter for systemic physiology and motion artifacts, and the data were converted to relative concentrations of oxygenated (HbO) and deoxygenated (HbR) blood using the modified Beer-Lambert law [55]. For each trial, the measured concentrations of HbO and HbR from −2 to 0 s were established as baseline for that trial and any changes in activation within that trial were compared to that baseline.

## 3. Results

### 3.1. Looking Time Analysis

Looking times were calculated for each 20 s trial, and a grand average was computed for the two speech conditions. Because the baseline consisted of a blank screen with no sound, looking times were not calculated for these periods. Test trials during which infants looked away from the screen for more than two consecutive seconds or for more than five seconds overall were eliminated from further analysis. No such trials existed. The average cumulative looking time during the English (native) condition was 16 s (SD = 0.62), while during the non-native (Spanish) condition it was 15 s (SD = 0.69), consistent with looking times observed in other NIRS research of this type [42,50,54,56,57]. Given the short run-time of the entire experiment (5 min), we did not anticipate that infants would develop expectations about the pattern of stimulus trials. Nonetheless, we also coded infants’ responses for anticipatory orientation towards the screen prior to each trial’s onset (during the pretrial baseline period). No instances of anticipatory orientation prior to trial onset were detected. Interrater-reliability of 97% was found between looking time calculations by the two observers; disagreements were reconciled through discussion.

### 3.2. Hemodynamic Analyses

Trials objectively categorized as containing motion artifacts (a change in the filtered intensity greater than 5% in 1/20 s during the 10 s baseline and 20 s test event) were eliminated from these analyses [54]. A total of 38 trials were eliminated due to motion artifacts. Two additional trials were eliminated because the infants failed to watch the event. A total of 9 trials were eliminated due to procedural error. In total, 49 trials out of 350 were eliminated and not included in our dataset. On average, 2 trials were excluded for each infant out of 10 possible trials (Mean = 2.24, SD = 1.48).

Relative concentrations of HbO obtained between 5 and 20 s following initiation of each trail were compared to that trial’s own baseline (measured from −2 s to trial onset). Because the hemodynamic response is still being initiated between 0 and 5 s, that time was not included in the average. Average changes in HbO concentration were calculated for each cortical region, measured by each of the four channels during each of the stimulus-specific trials relative to that trial’s own baseline. Although mean value for relative changes in concentration of both HbO and HbR were calculated (see Supplementary Tables S1 and S2), analyses were limited to HbO, as this chromophore generally provided the most robust contrast-to-noise ratio. 

To examine how levels of HbO changed over time during exposure to native and non- native speech in the 4 temporal regions of interest, a repeated-measures ANOVA was conducted. We excluded the first five seconds of data from analyses because infants’ hemodynamic responses take approximately that long to fully manifest. Time points 6 through 20 s were included in this analysis, yielding 15 time points. We selected a repeated-measures ANOVA because it is better suited to handle multiple samples across time than a multivariate ANOVA. Finally, we observed the values for Mauchly’s Test of Sphericity and used (Greenhouse-Geisser) corrections when necessary to account for differences in variances between time points.

Based on a 2 (Condition: non-native, native) × 4 (Channel: 1 = right anterior temporal, 2 = right posterior temporal, 3 = left anterior temporal, 4 = left posterior temporal) × 15 (Time: time points per trial) × 3 (Between subject factor, Age: 3-to-6-month-olds, 7-to-10-month-olds, 11-to-14-month-olds) repeated-measures, mixed ANOVA, the following results were obtained. 

### 3.3. Main Effects

A main effect of Condition, *F*(1, 1) = 19.95, *p* < 0.001, indicated that larger hemodynamic responses were elicited from infants in response to native compared with non-native speech when averaged across age. A main effect of Channel, *F*(1, 3) = 14.5, *p* < 0.001, indicated that, overall, infants showed different hemodynamic responses across the four measurement channels. Both channels on the left temporal area, in addition to the right anterior channel showed an increase in HbO during stimulus presentation. Only the right posterior channel showed a decrease in HbO during stimulus presentation. Finally, a main effect of Age, *F*(1, 2) = 4.29, *p* < 0.01, indicated that, regardless of speech condition, infants’ processing patterns increased significantly with age.

### 3.4. Interactions

**Figure 2:**
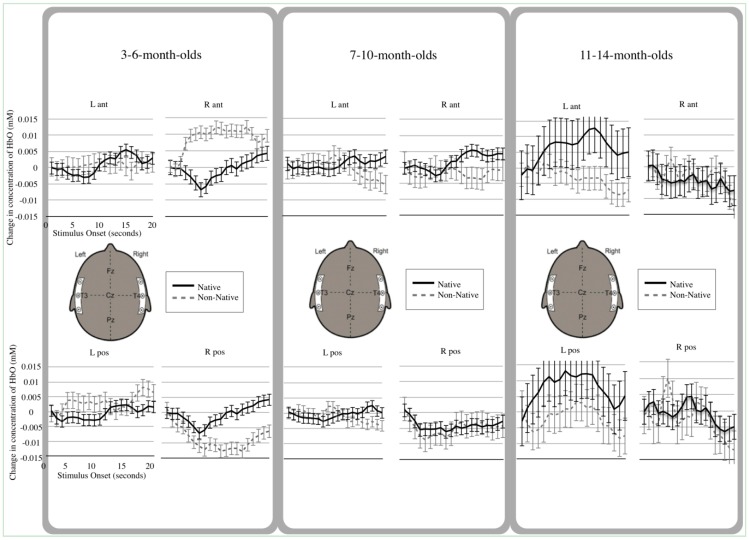
Hemodynamic Response Functions. Native speech is plotted in black, Non-Native speech is plotted in dashed gray. Each channel location is shown: Right Anterior (R ant), Right Posterior (R pos), Left Anterior (L ant) and Left Posterior (L pos). Error bars represent standard error of the estimate. Data from 3-to-6-month-olds are in the left panel; those from 7-to-10-month-olds are in the middle panel, and those from 11-to-14-month-olds are in the right panel.

Analyses also revealed several two-way interactions, as well as a three-way interaction among Condition, Channel, and Age. The first two-way interaction between Condition and Age showed that, depending on their age, infants produced different hemodynamic responses when listening to native and non-native speech, *F*(1, 2) = 4.36, *p* < 0.01 (see Figure 2). Degrees of freedom in the Bonferroni-corrected paired *t*-tests reported below reflect 15 time points per infant, 4 cortical locations of measurement, and the total number of infants per age group. *t*-tests revealed that the oldest group of infants (11-to-14-month-olds) produced significantly greater hemodynamic activity in response to native speech compared to non-native speech, *t*(479) = 4.27, *p* < 0.0001. In contrast, no significant differences emerged in the two younger groups of infants (3-to-6-month-olds, *t*(839) = 0.43, *p* = 0.67; 7-to-10-month-olds, *t*(779) = 1.42, *p* = 0.16) when comparing average response to native and non-native speech. 

The second 2-way interaction, between Channel and Age, indicated that infants of different ages differentially utilized the four cortical regions of interest during speech processing, *F*(1, 6) = 15.85, *p* < 0.001. Bonferroni-corrected paired *t*-tests showed that, in the right anterior channel, both 3-to-6-month-olds, and 7-to-10-month-olds had greater levels of activation, compared with 11-to-14-month-olds, (*t*(239) = 2.88, *p* < 0.005 and, *t*(239) = 5.41, *p* < 0.001, respectively). In the posterior left channel, 3-to-6-month-olds had higher levels of HbO compared with 7-to-10-month-olds, *t*(389) = 3.32, *p* = 0.001.

Finally, the 2-way interaction between Condition and Channel demonstrated that, averaging across age, regional responses varied significantly given the two different speech conditions, *F*(1, 3) = 3.34, *p* < 0.05. Bonferroni-corrected paired *t*-tests showed greater activation was observed overall in the left anterior channel for native compared to non-native speech, *t*(524) = 4.88, *p* < 0.001; in contrast, greater activation was observed overall in the right anterior channel for non-native compared to native speech, *t*(524) = 3.06, *p* < 0.05.

Importantly, the 3-way interaction of Condition, Channel, and Age, *F*(1, 6) = 8.38, *p* < 0.001, qualifies the main effects and 2-way interactions thus outlined. Bonferroni-corrected paired *t*-tests showed that in the right anterior channel only 3-to-6-month-olds showed a significantly greater activation for non-native compared with native speech, *t*(209) = 5.28, *p* < 0.001.

For the left anterior channel, only 11-to-14-month-olds showed significantly greater activation for native compared with non-native speech, *t*(119) = 3.327, *p* = 0.001. In the left posterior channel, all three groups showed significant differences in hemodynamic response to native and non-native speech. Three-to-six-month-olds were unique in that they showed a hemodynamic pattern reversed relative to the other two age groups in that region (non-native > native), *t*(209) = −3.18, *p* < 0.005. Both 7-to-10- and 11-to-14-month-olds showed the opposite pattern of activation (native > non-native), *t*(194) = 4.16, *p* < 0.001 and, *t*(119) = 3.33, *p* = 0.001, respectively. 

## 4. Discussion

The goal of the present study was to track infants’ cortical responses to both familiar and unfamiliar continuous audiovisual speech across the first year and a half of life. Given our engaging audiovisual stimuli, we expected to observe differences in hemodynamic responses to native and non-native speech in even the youngest infants, as well as the emergence of an increasingly (left) lateralized response to native relative to non-native speech before the age of 12-to-14 months [45,47].

### 4.1. Young Infants’ Differential Native and Non-Native Speech Processing

First, we observed differential hemodynamic responses to native and non-native speech before the age of four months. Indeed, 3-to-6-month-olds processed native and non-native speech differentially, and they were the only group of infants to produce a greater overall response to non-native compared to native speech. As noted, a difference in response volume to native and non-native speech has been observed by other researchers in four-month-olds [49], although the specific pattern was reversed (*i.e.*, native > non-native) from that observed in the present study (*i.e.*, native < non-native). These differences are likely due to disparities in the stimuli used in the two studies. Where Minagawa-Kawai and colleagues [49] used toys to capture infants’ attention while auditory-only stimuli (taken from film dialogues and including by both male and female speakers) played in the background, we presented infants with audiovisual infant-directed speech in the two languages from the same (female) speaker. It is likely that our stimuli engaged infants’ attention differently than those used in the previous study, thereby influencing which aspects of the signal infants attended to most. The disparity in results likely stems from the familiarity (not to mention ecological validity) of seeing a talking face producing speech; in this familiar context, the novelty of the unfamiliar speech may have boosted infants’ attention to the prosodic aspects of the signal.

To couch these findings in theoretical terms, it is possible that the involvement of the right anterior area is unique to 3-to-6-month-olds because they focus more on the spectral components of the signal (e.g., prosodic changes; emotional information) that typically engage right hemisphere processing [58]. The right hemisphere’s involvement in processing spectral aspects of speech, particularly in the case of suprasegmental cues, has been well documented using NIRS [40,41,59]. Another possibility is that the social novelty of the non-native speech was more salient for younger compared with older infants, and this increase in attention resulted in the recruitment of a wider range of cortical networks.

### 4.2. Older Infants’ Use of the Left Anterior Temporal Area

Although the 7-to-10-month-olds showed significant activation in the left posterior area for native compared with non-native speech, only the oldest infants showed a lateralized response to native compared with non-native speech in both the anterior and posterior regions of the left hemisphere.

Engagement of both anterior and posterior left temporal regions when processing native compared with non-native speech could be unique to 11-to-14-month-olds because they are tuned to the specific sounds within the native language. This relies on the identification of rapid temporal changes in the signal compared with those slower spectral cues relevant to processing prosody. The overall increase in hemodynamic activity in the oldest group of infants in response to native compared to non-native speech indicates that they were generally more engaged by this familiar form of speech (in contrast to the younger infants), although given the regions of activation observed, older infants’ focus of attention appears to have been on finer grained aspects of the speech signal (e.g., phonological structure) than that observed in the younger infants. Although our results indicate that all infants (from 3-to-14-months) engaged the left hemisphere while processing the two different types of audiovisual speech, the younger groups utilized only the posterior left region while the oldest group showed activation in both the anterior and posterior left temporal regions. We propose that increased recruitment of the left anterior region could be an indicator of the emergence of mature temporal processing of the sort that distinguishes among native (compared with non-native) speech sounds.

### 4.3. Interpretation of Deactivated Hemodynamic Functions

Seven-to-ten-month-olds showed a deactivation in hemodynamic response to both forms of speech in the right posterior region. Although deactivations of this sort have been reported in several studies using NIRS (see [60]), there is little consensus about what these decreases actually mean [61]. Some have proposed that they correspond to a decrease in the activity of large neural populations [62], whereas others suggest a reduction in response due to redistribution of blood flow [63]. In this case, “redistribution” refers to a measure of global blood flow that is not specific to a particular, localized area of activity (*i.e.*, the “blood steal” phenomenon) [63]. Still, others propose that such deactivations represent inhibitory neural connections [64], or an immature vascular system in the developing brain [65].

In the context of the four targeted cortical regions, it is difficult to pinpoint the root of the observed decreases in oxygenated hemoglobin, as we did not collect data from other cortical areas where corresponding increases in activation may have occurred. Regardless, it is improbable that the deactivation affected measurements in our other target regions, since both animal models (e.g, [66,67]) and human fMRI data (e.g., [68]) show that it is unlikely for deactivations to cause a general change in direction of blood flow (e.g., posterior to anterior). Indeed, several mechanisms and structures within the brain regulate this process (e.g., [69]). Future NIRS studies using whole-head probes will be needed to examine this issue in more detail.

Finally, while we employed both audio and visual speech streams to keep the wide age range of infants included in this study engaged with the stimuli, future work will need to determine how much the visual component of the audiovisual signal augmented infants’ auditory processing. Although we are not concerned that our focal regions of interest (*i.e.*, probes centered over T_3_/T_4_) overlapped with regions demonstrated to be active specifically to the visual component of audiovisual speech in fMRI studies of children (e.g., [70,71]) and adults [71,72], we are currently examining the relative involvement of those regions in infants while they process the components of audiovisual speech alone and in combination to better understand whether or not this is the case.

## 5. Conclusions

The present study dissociated which cortical regions are most engaged by infants ranging in age from 3 to 14 months while they are exposed to continuous audiovisual speech in their own or another language. Our findings highlight the fact that patterns of hemodynamic activity change markedly and distinctly in response to the two forms of speech over the course of the first year of life. Overall, younger infants recruited right hemisphere regions while older infants showed strongly left lateralized processing. Older infants responded most strongly to native compared with non-native speech, while younger infants showed the opposite pattern of cortical activity. At present, we cannot isolate the relative influence of the speech stimuli themselves, the role of social cues (e.g., via the talking face), increasing experience with language, and general biological maturation on these changing processing patterns. What is clear from our data is that continuous audiovisual speech is an engaging stimulus that allows the same experiment to be conducted with infants across a wide range of ages. Given that infants encounter such speech in their everyday lives, it is not surprising that they demonstrate dynamic means of processing it.

## Supplementary Files

Supplementary File 1
