# Supplementary material for: Dissociating Cortical Activity during Processing of Native and Non-Native Audiovisual Speech from Early to Late Infancy
Source: Brain Sci. 2014 Aug 11;4(3):471–87. doi: 10.3390/brainsci4030471 (PMC4194034; doi:10.3390/brainsci4030471)
Supplement: Supplementary File 1 [file brainsci-04-00471-s001.pdf]

Supplementary Information

Table S1. Changes in relative concentration of HbO following initiation of the test event, by neural region and stimulus condition. Cells contain μM (SE) μMolar cm averaged from 5 to 20 s.

Neural Region	Stimulus Condition	Age Group		
		**3–6 Months**	**7–10 Months**	**11–14 Months**
Right Anterior	Native	−0.58 (0.55) *	2.37 (0.76) +	−5.37 (0.66)
	Non-Native	10.40 (0.33)	−1.61 (0.35)	−3.64 (1.19)
Right Posterior	Native	−1.37 (0.87) +	−4.36 (0.29)	−1.52 (1.23)
	Non-Native	−10.3 (0.63)	−5.13 (0.45)	−2.79 (1.64)
Left Anterior	Native	1.87 (0.80) +	1.22 (0.40)	7.99 (1.00) *
	Non-Native	1.11 (0.31)	−0.74 (0.85)	−3.80 (0.85)
Left Posterior	Native	0.13 (0.60) **	−0.35(0.41) *	8.71 (1.37) *
	Non-Native	3.84 (0.72)	−1.44 (0.51)	−1.00 (0.99)

* Native versus Non-Native values within this cell are significantly different, $p \leq 0.001$.; ** Native versus Non-Native values within this cell are significantly different, $p < 0.05$.; + The HbO Native and Non-Native values are significantly different from each other, but do not remain significant. After correcting for multiple comparisons. As such, these differences are not discussed in the manuscript.

Table S2. Changes in relative concentration of HbR concentration following initiation of the test event, by neural region and condition. Cells contain μM (SE) μMolar cm averaged from 5 to 20 s.

Neural Region	Stimulus Condition					
	**Native Speech**			**Non-Native Speech**		
	**3–6 Months**	**7–10 Months**	**11–14 Months**	**3–6 Months**	**7–10 Months**	**11–14 Months**
Right Anterior	−0.25 (0.23)	−0.80 (0.27)	−0.43 (0.67)	−1.22 (0.14)	2.18 (0.20)	−3.92 (0.51)
Right Posterior	0.83 (0.19)	−2.55 (0.38)	1.34 (0.44)	−1.52 (0.29)	−4.47 (0.28)	1.92 (0.52)
Left Anterior	0.79 (0.18)	0.00 (0.16)	−0.09 (0.62)	−2.30 (0.24)	−0.03 (0.16)	−0.83 (0.39)
Left Posterior	−3.20 (0.42)	1.86 (0.18)	−0.19 (0.80)	−3.62 (0.82)	0.44 (0.27)	0.36 (0.55)

© 2014 by the authors; licensee MDPI, Basel, Switzerland. This article is an open access article distributed under the terms and conditions of the Creative Commons Attribution license (http://creativecommons.org/licenses/by/3.0/).
